# Causal relationship between gut microbiota and dental caries: a two-sample mendelian randomization study

**DOI:** 10.1038/s41405-025-00328-6

**Published:** 2025-04-10

**Authors:** Yang Wang, Quan Li, Jinqi Hua, Hongliang Que, Haoxiang Xu, Xinyu Xu, Ninghan Feng

**Affiliations:** 1 Department of urology, Jiangnan University Medical Center (Wuxi No. 2 People’s Hospital), Wuxi, 214000 China; 2 Department of urology, The Affiliated Suzhou Hospital of Nanjing Medical University, Suzhou, China

**Keywords:** Oral diseases, Dental conditions

## Abstract

**Background:**

In recent years, an increasing number of studies have revealed a close relationship between the gut microbiota and a variety of human diseases. At the same time, it has also been shown that dysregulation of the oral microbiota may lead to changes in the gut microbiota. However, it remains unclear whether the gut microbiota affects the occurrence and development of oral diseases. Therefore, the aim of this study was to explore the potential effects of gut microbiota on dental caries and to reveal possible mechanisms of the gut-oral microbiota axis.

**Methods:**

First, gut microbiota and dental caries data from genome-wide association studies (GWAS) were analyzed using Mendelian randomization analysis. Inverse variance weighted (IVW) was used as the main criterion (*P* value < 0.05). Then, MR-Egger regression, IVW regression and leave-one-out tests were used to test the reliability and stability of the mendelian randomization results. Finally, the potential mechanisms and significance of the relationship between gut microbiota and dental caries were explored.

**Results:**

The analysis showed that *Eubacteriumbrachygroup* [odds ratio (OR) = 1.001, 95% confidence interval (CI): 1.000–1.002, *P* = 0.046] and *Terrisporobacter* (OR = 1.002, 95% CI: 1.0001–1.0041, *P* = 0.035) were positively correlated with dental caries. *Escherichia.Shigella* (OR = 0.997, 95% CI: 0.995–0.999, *P* = 0.047), *Oscillibacter* (OR = 0.998, 95% CI: 0.997–0.999, *P* = 0.038), *RuminococcaceaeUCG014* (OR = 0.998, 95% CI: 0.996–0.999, *P* = 0.044) and *Oscillospira* (OR = 0.997, 95% CI: 0.995–0.999, *P* = 0.038) were negatively correlated with dental caries.

**Conclusion:**

The present study demonstrated a significant causal relationship between the gut microbiota and the development of dental caries, providing new insights into influencing the development of dental caries by affecting the composition of the gut microbiota.

## Introduction

Dental caries is one of the most common chronic oral diseases and has a significant impact on an individual’s quality of life [1] According to the fourth national oral health survey in China, the prevalence of dental caries among five-year-old children was 71.9%, while among 12-year-olds, it was 38.5% [2]. In the current etiology, dental caries occurs as a result of multifaceted interactions between microorganisms and various compounds (e.g., carbohydrates) [1]. Therefore, prevention of dental caries is crucial.

With the increased prevalence of dental caries, there is a growing interest in the impact of the gut microbiota on oral health. Gut microbiota are microorganisms, including bacteria, fungi, viruses, and archaea, that are designated to colonize the host’s intestinal tract [3]. The gut microbiome is now regarded as the second brain of the human body due to its remarkable diversity and the pivotal role it plays in maintaining host health and preventing disease [4]. In addition, the host’s diet and routine also have an impact on the abundance of gut microbiota, and this interdependent symbiotic relationship influences host physiological functions [5]. Recent research has demonstrated that the effects of gut microbiota on the host are involved in a number of processes, including human growth and development, metabolism, immunity, and pathophysiological processes [4, 6]. The human gut contains a large number of various microorganisms such as bacteria, fungi, viruses and archaea that influence the state of health of the host [7]. Lam, Gretchen A et al. summarized the association between gut microbiota disorders and periodontal disease, suggesting a potential correlation between gut microbiota and oral disease [8]. The beneficial gut microbiota can aid in the prevention of oral diseases. Several studies have shown that probiotic supplementation of gut microbiota is linked to decreased inflammation in periodontitis and dental caries [9–11]. This may be associated with increased metabolites in the gut microbiota, such as short-chain fatty acids. Previous studies have demonstrated that supplementation with short-chain fatty acids can be effective in combating systemic inflammation [12]. Additionally, changes in the abundance of gut microbiota can enhance fluoride absorption, subsequently contributing to the prevention of dental caries [13, 14]. Increasing research has shown that gut microbiota dysbiosis can promote systemic inflammation in the body. It was found that gut microbiota dysbiosis leads to the introduction of lipopolysaccharide (LPS) into the circulatory system, which ultimately leads to systemic inflammation [15, 16]. And, it has also been shown that LPS can contribute to the development of dental caries by affecting pulp stem cell activity [17, 18]. Thus, there is a clear correlation between gut microbiota and risk of dental caries development, but observational studies are susceptible to confounding by confounding factors leading to biased results.

Mendelian randomization (MR) represents a highly promising approach that is designed to circumvent the potential for bias in observational epidemiology. The method employs an examination of the correlation between the degree of genetic prediction of exposure factors (e.g., gut microbes) and disease outcomes (e.g., dental caries). The three key assumptions of mendelian randomization aim to minimize confounding variables and enhance the ability to establish causal relationships compared to traditional epidemiological studies. The existing literature extensively examines the association between gingival microbiota and periodontitis; however, the causal relationship between gut microbiota and dental caries remains unclear. This study aims to explore the potential causal link between gut microbiota and dental caries, shedding light on the role of gut microbiota in the development of dental caries and potential therapeutic approaches.

## Materials and methods

### Study design

Mendelian randomization is contingent upon three fundamental assumptions: (1) The instrumental variable (IV) must be strongly associated with the exposure factor; (2) the instrumental variables must not be affected by confounders; and (3) the instrumental variables must affect the outcome only through the exposure factor [19]. In this study, we employed gut microbiota GWAS data as an exposure factor and dental caries GWAS data as an outcome. In accordance with the established inclusion criteria, we selected appropriate single-nucleotide polymorphisms (SNPs) as instrumental variables and conducted a two-sample mendelian randomization analysis to investigate the potential causal relationship between the gut microbiota and dental caries. Similarly, this study adhered to the STROBE-MR guidelines in a rigorous manner [20].

### Sample data sources

The GWAS data on the gut microbiota were derived from a comprehensive meta-analysis conducted by the MiBioGen consortium (www.mibiogen.org). This is the most extensive, multi-ethnic, genome-wide meta-analysis of the gut microbiota to date, which analyzed genome-wide genotyping data and 16S fecal microbiota data from 24 cohorts (18,340 individuals). For more detailed information, please refer to the original study [21]. The majority of participants surveyed were of European ancestry (N = 13,266), and this study primarily involved 131 genera. The direct taxonomic binning method was used to classify the microbiota and performed microbial quantitative trait locus (mbQTL) analysis to identify host genetic variation loci associated with the abundance levels of bacterial taxa in the gut [22]. Genus was the smallest taxonomic level in this analysis, and the study identified 131 genera with mean abundances greater than 1%. We analysed data from a total of 131 genera of European origin in this study to analyse the causal relationship between gut microbes and dental caries. Dental caries data were obtained from the GWAS Summary data set, which was obtained from the IEU Open GWAS project (https://gwas.mrcieu.ac.uk/).

### Instrumental variables selection

The bacterial taxa were classified and analyzed at the genus level. To guarantee the accuracy and validity of conclusions about the causality between gut microbiota and dental caries risk, the following quality control procedures were applied to filter instrumental variables. Given the limited number of instrumental variables obtained when the threshold was set to (*P* < 5 × 10^−8^), we elected to select SNPs using a threshold of (*P* < 1 × 10^−5^) in order to obtain a greater number of instrumental variables and more reliable results. Secondly, to account for linkage disequilibrium and avoid biased results, we set the linkage disequilibrium parameter (R^2^) for SNPs at 0.001 and the genetic distance at 10,000 kb [22]. This ensured that each instrumental variable was independently present. We set the minor allele frequency level at 0.01 and excluded palindromic SNPs and those that were not present in the outcome. Given that instrumental variable is an instrumental variable, it is necessary to ascertain whether there is a strong correlation between the instrumental variable and exposure. To this end, the F-statistic was employed to assess the correlation between the instrumental variable and exposure. It is our contention that an F-statistic exceeding 10 signifies a robust correlation between the two variables.

### Mendelian randomization analysis

In this study, we employed efficacious methodologies, including inverse variance weighting (IVW), MR-Egger, weighted median, and weighted mode. The IVW method represents the principal approach utilized in mendelian randomization analysis. This method employs meta-analysis to synthesize the Wald estimates associated with each individual SNP, thereby producing an overall estimate of the collective impact of gut microbiota on dental caries. If causality can be established by IVW methods (*P* < 0.05), the IVW results are complemented by efficient methods such as MR-Egger, weighted median, and weighted mode. The MR-Egger method does not force the intercept to be zero, allowing for the estimation of causal effects even in the presence of null instruments (SNPs that can influence the results through non-exposure pathways). Furthermore, the intercept can indicate the degree of horizontal pleiotropy [23]. This two-sample MR analysis was performed using R software (version 4.4.0) with TwoSampleMR (version 0.5.6) and MR-PRESSO packages (version 1.0.0).

## Result

The results of the mendelian randomization analysis identified 6 caries-related bacterial genera (*Escherichia.Shigella*, *Oscillibacter*, *Eubacteriumbrachygroup*, *Terrisporobacter*, *RuminococcaceaeUCG014*, and *Oscillospira*) and the results of the IVW method demonstrated significant differences (*P* < 0.05). IVW results showed that *Eubacteriumbrachygroup* [odds ratios (OR) = 1.001, 95% confidence interval (CI): 1.000–1.002, *P* = 0.046] and *Terrisporobacter* (OR = 1.002, 95% CI: 1.0001–1.0041, *P* = 0.035) were positively associated with dental caries. IVW results showed that *Escherichia.Shigella* (OR = 0.997, 95% CI: 0.995–0.999, *P* = 0.047), *Oscillibacter* (OR = 0.998, 95% CI: 0.997–0.999, *P* = 0.038), *RuminococcaceaeUCG014* (OR = 0.998, 95% CI: 0.996–0.999, *P* = 0.044), and *Oscillospira* (OR = 0.997, 95% CI: 0.995–0.999, *P* = 0.038) were negatively associated with dental caries (Table 1).

**Table 1:** Summary results of mendelian randomization (Target gut microbiota on dental caries).

					Cochran Q-test		Directional pleiotropy	
Exposure	Method	NSNPs	OR (95% CI)	*P*	*P*	I^2^ (%)	Egger intercept (*P*)	Correct Causal direction
*Escherichia.Shigella*	IVW	7	0.997 (0.995–0.999)	0.047	3.69E-01	0.92	1.10E-03	TRUE
*Escherichia.Shigella*	MR-Egger	7	0.980 (0.961–0.999)	0.101				
*Escherichia.Shigella*	Weighted median	7	0.998 (0.995–1.000)	0.183				
*Escherichia.Shigella*	Simple mode	7	0.999 (0.994–1.003)	0.658				
*Escherichia.Shigella*	Weighted mode	7	0.999 (0.995–1.002)	0.647				
*Oscillibacter*	IVW	9	0.998 (0.997–0.999)	0.038	8.07E-01	1.76	1.97E-04	TRUE
*Oscillibacter*	MR-Egger	9	0.996 (0.977–1.015)	0.703				
*Oscillibacter*	Weighted median	9	0.998 (0.996–1.000)	0.050				
*Oscillibacter*	Simple mode	9	0.997 (0.995–1.000)	0.122				
*Oscillibacter*	Weighted mode	9	0.997 (0.994–1.000)	0.164				
*Eubacteriumbrachygroup*	IVW	6	1.001 (1.000–1.002)	0.046	9.09E-01	3.25	-7.17E-05	TRUE
*Eubacteriumbrachygroup*	MR Egger	6	1.001 (0.982–1.021)	0.867				
*Eubacteriumbrachygroup*	Weighted median	6	1.000 (0.999–1.002)	0.132				
*Eubacteriumbrachygroup*	Simple mode	6	1.000 (0.998–1.002)	0.404				
*Eubacteriumbrachygroup*	Weighted mode	6	1.000 (0.999–1.002)	0.399				
*Terrisporobacter*	IVW	3	1.002 (1.0001–1.0041)	0.035	3.73E-01	1.01	1.78E-03	TRUE
*Terrisporobacter*	MR-Egger	3	0.980 (0.951–1.011)	0.429				
*Terrisporobacter*	Weighted median	3	1.002 (0.999–1.004)	0.118				
*Terrisporobacter*	Simple mode	3	1.002 (0.999–1.006)	0.278				
*Terrisporobacter*	Weighted mode	3	1.002 (0.999–1.005)	0.292				
*RuminococcaceaeUCG014*	IVW	10	0.998 (0.996–0.999)	0.044	1.75E-01	0.70	9.83E-05	TRUE
*RuminococcaceaeUCG014*	MR-Egger	10	0.996 (0.972–1.020)	0.78				
*RuminococcaceaeUCG014*	Weighted median	10	0.999 (0.997–1.001)	0.774				
*RuminococcaceaeUCG014*	Simple mode	10	1.000 (0.996–1.003)	0.939				
*RuminococcaceaeUCG014*	Weighted mode	10	1.000 (0.997–1.003)	0.948				
*Oscillospira*	IVW	4	0.997 (0.995–0.999)	0.038	7.71E-01	2.66	9.08E-04	TRUE
*Oscillospira*	MR-Egger	4	0.986 (0.953–1.020)	0.509				
*Oscillospira*	Weighted median	4	0.997 (0.995–1.000)	0.06				
*Oscillospira*	Simple mode	4	0.996 (0.993–1.000)	0.173				
*Oscillospira*	Weighted mode	4	0.996 (0.993–1.000)	0.181				

*MR* Mendelian randomization, *NSNPs* number of single-nucleotide polymorphisms, *OR* odds ratio, *CI* confidence interval, *IVW* inverse variance weighting.

**Fig. 1:**
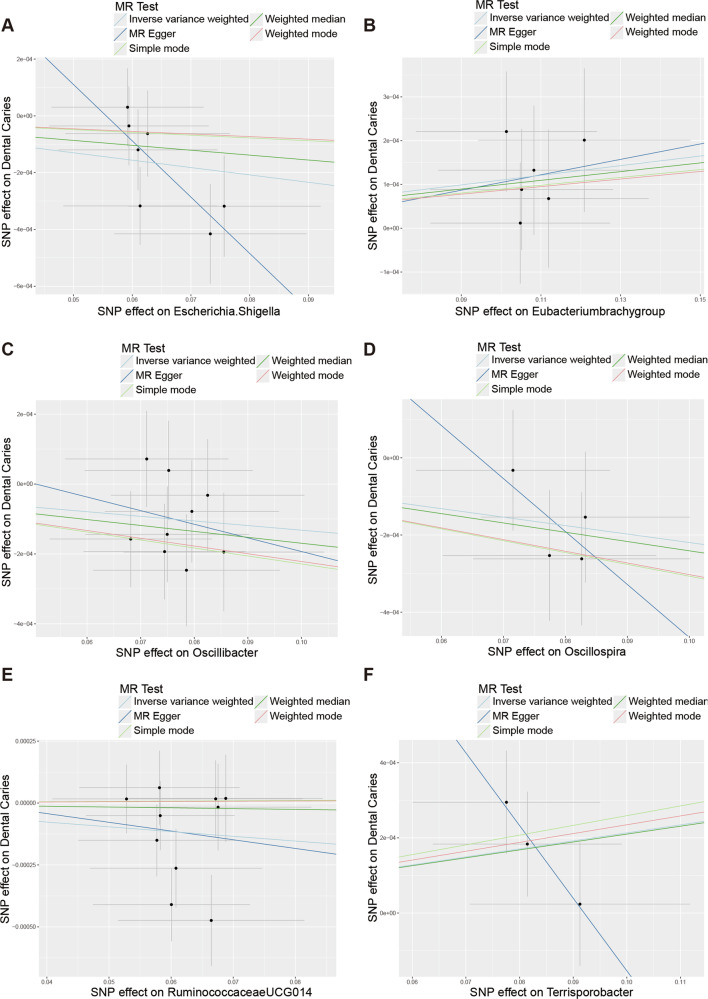
The scatter plots illustrate the significant causal relationship between the gut microbiota and dental caries. The scatter plot of the effect size and 95% confidence interval of each SNP on gut microbiota and dental caries risk demonstrates the impact of each SNP on the risk of dental caries. The horizontal axis reflects the genetic effect of each SNP on gut microbiota, while the vertical axis represents the genetic effect of each SNP on dental caries risk. **A** Causal relationship between dental caries and *Escherichia.Shigella*. **B** Causal relationship between dental caries and *Eubacteriumbrachygroup*. **C** Causal relationship between dental caries and *Oscillibacter*. **D** Causal relationship between dental caries and *Oscillospira*. **E** Causal relationship between dental caries and *RuminococcaceaeUCG014*. **F** Causal relationship between dental caries and *Terrisporobacter*.

**Fig. 2:**
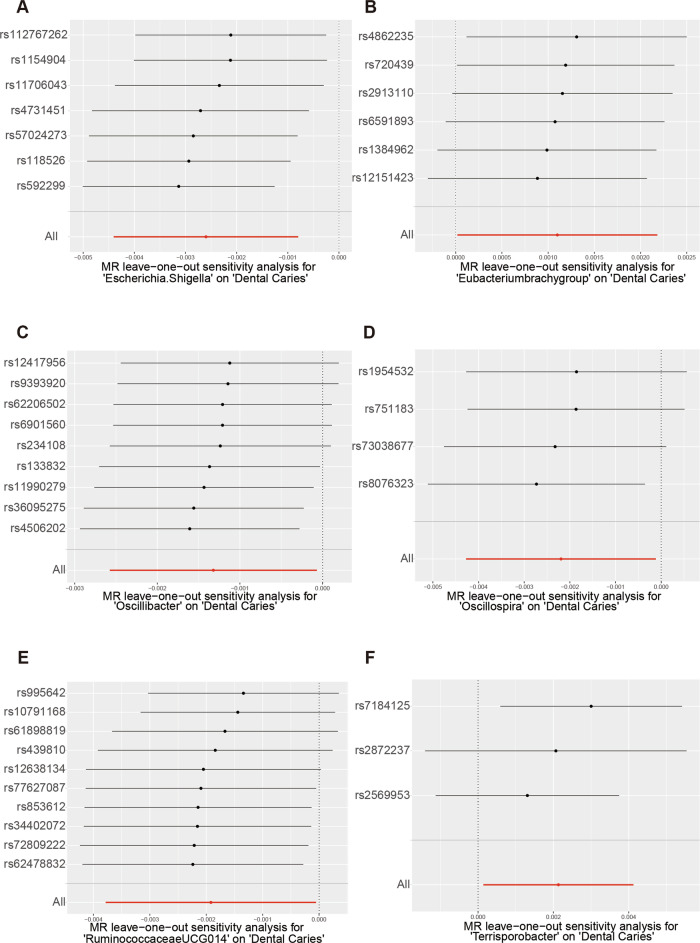
Leave-one-out analysis for the impact of individual SNPs on the association between gut microbiota and dental caries risk. By leaving out exactly one SNP, it demonstrates how each individual SNP influences the overall estimate. **A** Leave-one-out plot of the causal relationship between dental caries and *Escherichia.Shigella*. **B** Leave-one-out plot of the causal relationship between dental caries and *Eubacteriumbrachygroup*. **C** Leave-one-out plot of the causal relationship between dental caries and *Oscillibacter*. **D** Leave-one-out plot of the causal relationship between dental caries and *Oscillospira*. **E** Leave-one-out plot of the causal relationship between dental caries and *RuminococcaceaeUCG014*. **F** Leave-one-out plot of the causal relationship between dental caries and *Terrisporobacter*.

The results of Cochran’s Q-test indicated that there was no heterogeneity between the instrumental variables (Table 1). In the horizontal polytomous test, we chose IVs that did not have horizontal polytomous validity,and any IVs that did not meet horizontal polytomous validity were excluded (*P* < 0.05) (Table S6). Scatterplot results showed a gradual decrease in the risk of caries development with increasing abundance of *Escherichia.Shigella*, *Oscillibacter*, *Oscillospira*, and *RuminococcaceaeUCG014* leading to a gradual decrease in the risk of caries development (Fig. 1A, C–E). And with the increase in abundance of *Eubacteriumbrachygroup*, *Terrisporobacter* led to increased risk of caries development (Fig. 1B, F). The results of the leave-one-out method showed the reliability of the mendelian randomization analysis results (Fig. 2). Furthermore, the results of all Steiger directionality tests demonstrated a robust directionality between gut microbiota and caries (Table S1).

## Discussion

The aim of this research was to investigate the potential causal association between gut microbiota and dental caries through a two-sample mendelian randomization analysis. This study presents the first large-scale mendelian randomization analyses exploring the genetic correlation between gut microbiota and dental caries, utilizing the latest and most extensive GWAS data available. The outcomes of these analyses could offer a theoretical basis for the development of strategies for the prevention, treatment, and prognostic management of dental caries. The findings of this study indicate that *Eubacteriumbrachygroup* and *Terrisporobacter* have a positive impact on the progression of dental caries, while *Escherichia.Shigella, Oscillibacter, RuminococcaceaeUCG014*, and *Oscillospira* have a negative impact on caries development.

The gut microbiota influences the pathogenesis of dental caries, and we prefer that the gut microbiota influences the development of dental caries by mediating metabolite alterations. A study by Yamada, Miki et al. reported that a metabolite of intestinal microorganisms (10-hydroxy-cis-12-octadecenoic acid) has a role in preventing tooth destruction [24]. In addition, bone metabolism has an important role in the development of dental caries. Short-chain fatty acids produced by Enterobacteriaceae can promote the differentiation and proliferation of osteoblasts, induce apoptosis of osteoclasts, and prevent the development of dental caries [25]. This suggests that dysbiosis of the gut microbiota can influence the progression of dental caries through changes in metabolites. *Escherichia.Shigella* is believed to be potentially associated with the development of dental caries, although there is currently no direct evidence of a causal relationship between the two [26, 27]. *Oscillibacter* is a member of the group of bacilli that are generally considered to be beneficial to the host [28, 29]. Although *Oscillibacter* has been less extensively studied, recent research has demonstrated its involvement in intestinal immunity and several metabolic processes, including fatty acid metabolism, polysaccharide degradation, and nitrogen metabolism [28, 30–32]. A robust correlation exists between polysaccharide metabolism and the development of dental caries [33]. Bacteria such as *Streptococcus mutans* are capable of converting polysaccharides into acidic metabolites, such as lactic acid, which can lead to enamel dissolution and thus facilitate the onset and progression of dental caries [34, 35]. *Oscillibacter* is capable of degrading complex polysaccharides, such as plant fibers and other indigestible carbohydrates [36]. These metabolic capabilities facilitate the provision of nutrients, such as short-chain fatty acids, which are essential for the proper functioning of intestinal cells. There is evidence that short-chain fatty acids have anti-inflammatory effects in the body. Furthermore, the metabolism of the gut microbiota affects the overall health of the host [37]. Consequently, *Oscillibacter* may be implicated in the pathogenesis of dental caries via the “gut-oral axis,” although further research is necessary to substantiate this hypothesis [38]. The relationship between *RuminococcaceaeUCG014* and *Streptococcus mutans* is primarily indirect, rather than direct. This is due to the fact that these two bacteria inhabit disparate biological environments and fulfill distinct biological functions and ecological roles. (1) Biological Environment: *Streptococcus mutans* is primarily found in the oral cavity, particularly in dental plaque, and is one of the most significant strains responsible for the development of dental caries. It is capable of utilizing carbohydrates present in food residues, and by producing acidic metabolites, it leads to acid erosion of the tooth surface, which ultimately results in the formation of dental caries [35]. (2) Functional and metabolic pathways: *Streptococcus mutans* is capable of adapting to the oral cavity, particularly in environments with low PH. They are capable of utilizing simple carbohydrates, such as glucose, and producing copious amounts of acidic metabolites, such as lactic acid, which is destructive to the mineralized layer of the teeth. (3) The role of *RuminococcaceaeUCG014*: In contrast, *RuminococcaceaeUCG014* are primarily found in the intestine and are involved in the breakdown of complex carbohydrates and the production of short-chain fatty acids [39]. They play a significant role in maintaining gut health and systemic metabolism, yet their activities do not directly intersect with the biological functions of the cariogenic bacterium *Streptococcus mutans* in the oral cavity. Although the overall state of the gut microbiota community may indirectly affect oral health through mechanisms such as regulation of the immune system, there is no evidence of direct metabolic cooperation or competition between *RuminococcaceaeUCG014* and *Streptococcus mutans*. Consequently, further studies are required to elucidate the specific interactions between these organisms and their impact on dental caries. The role of *Oscillospira* in oral diseases has been insufficiently investigated, yet it is now regarded as a promising probiotic candidate in the human body, with a potential inverse correlation with the onset of various diseases [40]. The associations between *Eubacteriumbrachygroup* and *Terrisporobacter* and the risk of caries development have not been extensively investigated.

Our research has limitations. Firstly, the results of this analysis are limited to a European population and may not be generalizable to other populations. Secondly, our study mainly explores the relationship between the two at the data level and is unable to conduct a clear mechanistic study. Finally, our study lacked the influence of individual dietary habits or other factors on the risk of caries development. Accordingly, further validation is required in future studies.

## Conclusions

In conclusion, causal relationships between the gut microbiota and dental caries were revealed through large-scale GWAS analyses. Among the identified bacterial genera, four exhibited a negative association with dental caries, while two demonstrated a positive association. Nevertheless, further investigation of the mechanism by which the gut microbiota influences dental caries is contingent upon the availability of a larger GWAS database.

## Supplementary information

Table S1

Table S2

Table S3

Table S4

Table S5

Table S6

## Data Availability

The raw data analyzed during the current study were available in public databases including IEU database (ukb-b-4770) and MiBioGen database (https://mibiogen.gcc.rug.nl). The code and data related to this study are available from the corresponding author upon reasonable request.
